# Knowledge and attitude on home-based management of diarrheal disease among mothers/caregivers of under-five children at a tertiary hospital in Ethiopia

**DOI:** 10.11604/pamj.2023.44.38.34431

**Published:** 2023-01-19

**Authors:** Bethelhem Shewangizaw, Mekdes Mekonen, Thomas Fako, Dawit Hoyiso, Yacob Abraham Borie, Tomas Yeheyis, Getinet Kassahun

**Affiliations:** 1 School of Nursing, College of Medicine and Health Sciences, Hawasa University, Hawasa, Ethiopia,; 2 Department of Midwifery, College of Medicine and Health Sciences, Hawasa University, Hawasa, Ethiopia

**Keywords:** Diarrheal disease, mothers/caregivers, knowledge, attitude, home-based management of diarrhea

## Abstract

**Introduction:**

diarrhea is the passage of three or more loose or liquid stools per day or more frequent than normal for the individual. Nearly half of deaths from diarrhea among young children occur in Africa where diarrhea is the largest reason for death among children under 5 years old. Home-based management of diarrhea plays its role in the treatment of a child with diarrhea. Hence, this study aims to assess knowledge and attitude on home-based management of diarrheal disease among mothers/caregivers of under-five children at a tertiary hospital in Ethiopia.

**Methods:**

an institutional-based cross-sectional study was conducted among 238 mothers/caregivers from October 21-November 21, 2021. Data was collected by using structured questionnaires containing 24 knowledge and attitude items then entered and analyzed using SPSS version 21. Bivariate and multivariable analyses were carried out to identify factors associated with the knowledge and attitude of mothers/caregivers towards home-based management of diarrhea using binary logistic regression. Statistical significance was set at p-value < 0.05.

**Results:**

the study revealed 36.6% of respondents had good knowledge and 55.5%of respondents had a favorable attitude towards home-based management of diarrhea. Being illiterate (Adjusted Odds Ratio (AOR)(95% confidence interval (CI) 0.123(0.027-0.554)) was significantly associated with the knowledge of mothers. Whereas being the mother (AOR(95%CI)3.085(1.071-8.890)) and having a monthly income < 2000birr (AOR(95%CI) 0.248(0.069-0.892)) had a significant association with the attitude of mothers/caregivers towards home-based management of diarrhea.

**Conclusion:**

unacceptable number of mothers/caregivers had poor knowledge regarding home-based management of diarrhea. Similarly, a high number of the respondents had a favorable attitude toward home-based management of diarrhea. Improving the educational status of mothers/caregivers by the educational sector and raising monthly income play a role in raising the knowledge and improving the attitude of mothers towards home-based management of diarrhea.

## Introduction

Diarrhea is defined as the discharge of three or more loose or liquid stools per day (or a discharge more frequent than normal for the individual) that lasts for several days and may result in a deficiency of water and salts that the body needs to survive [1]. The infection spreads through contaminated food or drink or from person to person as a result of poor hygiene. There are three clinical types of diarrhea: acute watery diarrhea lasting several hours or days, which includes cholera; acute bloody diarrhea (dysentery); persistent diarrhea lasting fourteen days or longer [1]. Acute diarrheal diseases are one of the most important problems for children in the world and therefore the second leading reason for preventable death, especially among children under five in developing countries. They affect children's well-being and result in significant demand for health services [2]. Worldwide, there are nearly 1.7 billion cases of diarrheal disease in children every year. It is estimated that there are 2.5 billion cases of illness and 1.5 million deaths in children under five years old every year [3]. That is, 21% of all deaths within the developing world, and also the number remains unacceptably high. Diarrhea kills a greater number of young children than acquired immunodeficiency syndrome (AIDS), malaria, and measles combined. It also exposes children to secondary infections [3].

In developing countries, one in ten newborns doesn´t reach his or her fifth birthday because he or she is a victim of diarrheal disease [3]. Eight out of ten deaths before five years of age occur in the first two years of life [3]. On average, children under three years of age experience diarrhea three times per year, with the incidence being highest in the first two years of life and decreasing as the child ages. The prevalence of diarrheal disease varies from place to place. Community practices regarding ORT and other treatment methods also vary from place to place [4]. However, the consequences of diarrhea in children often include dehydration, growth retardation, malnutrition, and impaired cognitive development [5]. In Africa, every child under five years of age suffers from diarrhea five times per year, and approximately 800,000 children die each year from diarrhea and dehydration [6]. Most of these deaths (42%) occur in sub-Saharan African countries [7].

In sub-Saharan Africa, primary caregivers know little about the signs of dehydration, dysentery, and the treatment of diarrheal disease [8]. Parents' and caregivers' attitudes toward managing the disease depend on how they assess the severity of the disease, especially in young children, and on how primary caregivers of children < 5 years access health care [9]. In Ethiopia, diarrheal disease affected approximately 13.3% of children under five years of age in 2012, according to EDHS [10]. Of these children, 3% had bloody diarrhea in the two weeks before the survey. Diarrhea was most common in children 6-23 months of age (23-25%). Diarrhea prevalence is highest among children living in households that drink from unprotected wells (18%) and among children living in rural areas (14%) [10]. This makes diarrhea the second leading cause of death among children after pneumonia and a serious public health problem. An estimated 73,700 children under the age of five die each year from diarrheal diseases. This accounts for an estimated 20% of under-five deaths in the country [11]. The family, especially the mother, plays a critical role in health promotion, disease prevention, and patient care. In the case of diarrheal illness, the minimum required of caregivers is a brief cursory examination and identification of the dehydrated child and the amount and type of fluids given for diarrhea, although these measures are critical to the well-being of children [12]. Although morbidity and mortality due to diarrheal illness have decreased significantly, there has been limited improvement in the home management of diarrheal illness in the general population [13]. However, home-based treatment of diarrheal diseases is inadequate among caregivers of children under five years of age, especially in developing countries, due to inadequate knowledge and attitudes [13].

Therefore, home-based treatment of diarrhea is quite common among mothers of children with uncomplicated diarrhea. Awareness and attitudes toward home management of diarrhea, as well as measures taken in the home to prevent or treat the disease, are critical to reducing morbidity and mortality associated with diarrhea. The result will hopefully give baseline data for any child health intervention to be implemented at Ethiopian health institutions, besides the result of this study will help clinicians to give attention to rising knowledge and attitude toward mothers of children with diarrhea. Therefore, this study investigates maternal knowledge and attitudes towards home management of diarrhea and associated factors among mothers/caregivers of children under five years of age attending Hawassa College Comprehensive Specialized Hospital, Hawassa, Ethiopia.

## Methods

**Study area:** the study was conducted at Hawassa University Comprehensive Specialized Hospital (HUCSH), Hawassa, southern Ethiopia. The hospital is found in the southwestern part of Hawassa town and is bordered to the east by Hawassa town, to the north by Tabor Mountain, to the west by Hawassa Lake, and the south by private residents. The hospital gives health services like inpatient and outpatient for about 15 million people from all over Southern Nations Nationalities and Peoples Region (SNNPR) and the neighboring Oromia region. Currently, it has 350 beds and provides patient care in a broad range of services on average to over 90, 200 outpatient, 18,116 hospitalized patients, and 1,092 emergency cases annually.

**Study design, and period:** an institution-based cross-sectional study was conducted from October 21-November 21, 2021 in Hawassa University Comprehensive Specialized Hospital (HUCSH), Hawassa, southern Ethiopia.

**Study population:** all sampled mothers/caregivers of under-five children seeking service in HUCSH during the study period were enrolled in the study. Mothers who were not volunteers were excluded from the study and replaced by the next mother/caregiver in the queue.

**Sample size determination:** the sample size was determined by using the single population proportion formula after fulfilling the following assumptions: 95% level of confidence, 5% margin of error, 56.2% proportion of good knowledge towards home-based management of diarrhea in Awi zone, Ethiopia [14]. Since the source population is less than 10,000 the reduction formula has been applied and after incorporating 10% non-response rate the final sample size of the study was 238.

**Sampling procedure:** Hawassa University Comprehensive Specialized Hospital is chosen for the study because it has a large catchment area, and provides service to children seeking service from the wide southern area of the country representing a larger community with a different culture. The average monthly number of children visiting under 5 services in the hospital was taken from records in the annual flow recorder of the hospital and then the average monthly number of children visiting under five OPD of the hospital (504) population was divided into the sample size of the study. This gives the interval at which study subjects will be selected. Based on this systematic random sampling was employed with an interval (K) of 2, meaning every other mother/caregiver was recruited for the study. The first respondent of the study was selected randomly from the first two clients.

**Data collection instrument and measurements:** the data collection instrument was prepared after reviewing different previous literature. The interviewer-administered questionnaire comprises socio-demographic information, socioeconomic status, knowledge assessment, and, attitude assessment. A pre-test was conducted on 5% of the sample size to ensure the validity of the tool, then corrections were made before the actual data collection. Thirteen knowledge assessment questions and 11 attitude assessment questions were used to assess the knowledge and attitude of mothers. Since the data were fairly distributed mean was used to demarcate good/poor knowledge and favorable/unfavorable attitude.

**Data collectors and data collection procedure:** a structured interviewer-administered questionnaire was prepared in English and translated into Amharic and vice versa to ensure its consistency. The one-day training was given to the 3 data collectors and 2 experienced supervisors on the tool and the data collection procedure. Data completeness, accuracy, and clarity were checked daily by the supervisors. The interview was conducted after the child gets the service and before leaving the hospital.

### Data processing and analysis

Data were coded, entered, and cleaned using Epi Data version 3.02 software. Errors related to inconsistency were verified using the data cleansing method. The entered data will be exported and analyzed with Statistical Package for Social Science (SPSS) version 24 software. Simple frequencies were done to see the overall distribution of the study participants with the different study variables. Descriptive statistics were used to summarize the socio-demographic characteristics of the study participants and the awareness of diarrhea management. To identify factors associated with diarrhea management, binary logistic regression analyses were carried out at two levels. To control confounding factors; variables with a p-value of 0.25 in the bivariate analysis, significant in previous studies, and biological plausibility were taken for the multivariable analysis. Standard error and Hosmer-Lemeshow tests were used to check multicollinearity and the model goodness of fit respectively. Adjusted odds ratio (AOR) with a 95% confidence interval (CI) was used to identify factors associated with the Knowledge and attitude of mothers/caregivers. The level of statistical significance was set at a p-value < 0.05. Multi-collinearity was checked to see the linear correlation among the independent variables by using the variance inflation factor and standard error. Variables with variance inflation factor >10 and standard error of > 2 were dropped from the multivariable analysis. A Hosmer-Lemeshow test was done to test for model fitness. The test resulted in a value > 0.05 and was taken as fit.

**Ethical consideration:** ethical clearance was obtained from Hawassa University, College of Medicine and Health Sciences Institutional Review Board. The participants were informed of the purpose of the study. Furthermore, before the commencement of the data collection written informed consent was obtained from all participants. Code numbers were used throughout the study to maintain anonymity and privacy was protected so that the respondents provide full information without fear of an intrusion. Confidentiality has been maintained during data collection and the collected data was confidentially stored in a secure area. The study has been conducted by respecting the Declaration of Helsinki.

## Results

**Socio-demographic and socio-economic characteristics:** a total number of 227 respondents have participated in the study giving a response rate of 95.3%. Of the study participants majority (56.4%) were between the ages of 25-34 years, a third (36.1%) were Protestant, one third (33.5%) of mothers/caregivers attended secondary school while one fifth (18.1%) received no formal education and nearly half (42.7%) were housewives (Table 1). Regarding the source of information majority (65.6%) of the respondents were TV owners 54.2% looked at TV frequently and half of the respondents (47.1%) listen to the radio frequently.

**Table 1:** socio-demographic variables of the mothers/caregivers in HUSCH, Hawassa, Ethiopia, 2021

Variable		Frequency (n=227)	Percentage (100%)
Age	15-24	57	25.1
	25-34	128	56.4
	35-44	20	8.8
	45 and above	22	9.7
Religion	Orthodox	62	27.3
	Muslim	73	32.2
	Catholic	10	4.4
	Protestant	82	36.1
Educational status	Illiterate	41	18.1
	Read and write	16	7
	Primary	59	26
	Secondary	76	33.5
	College	35	15.4
Occupation	Government employee	21	9.3
Relationship with child	Self-employee	58	25.6
	Housewife	97	42.7
	Merchant	51	22.5
	Mother	174	76.7
	Grandmother	28	12.8
	Caregiver	24	10.6
Marital status	Married	152	67
	Unmarried	22	9.7
	Divorced	25	11
	Widowed	28	12.3%
Monthly income	<2000	55	24.2
	2000-2999	90	39.6
	3000-3999	45	19.8
	>4000	37	16.3
Family size	<4	152	67
	>4	75	33

### Knowledge of mothers toward home-based management of diarrhea

Thirteen knowledge questions were asked to caregivers of under-five children about home-based management of diarrhea. Of 227 respondents majority (79.7%) correctly responded that hand washing is a diarrheal prevention method followed by breastfeeding and safe disposal of the stool of young children both of which account for 10.1%. More than half of respondents in the study (58.6%) do not know the signs of dehydration. According to this study, more than half of mothers/caregivers (55.5%) heard about oral rehydration solution (ORS) from health centers while (7.5%) and (4.8%) heard it from TV and radio respectively. Regarding the use of ORS 55.5% and 30.8% of the respondents correctly responded that it decreases diarrhea and replaces fluid lost respectively. On the other hand, 4.4% answered incorrectly as ORS increases diarrhea. The results of data were fairly distributed and the mean was used as an index for the classification of knowledge as good and poor. Of the total of 227 mothers/caregivers, 36.6% of them had good knowledge and 63.4% had poor knowledge.

### The attitude of mothers toward home-based management of diarrhea

Eleven attitude questions each scored out of five were asked to caregivers of under-five children about home-based management of diarrhea. Of the total respondents, nearly half (52.9%) of mothers/caregivers agreed that ORS is better than traditional medicine to manage diarrhea. About 37.9% of mothers/caregivers disagree that giving ORS and fluids aggravates diarrhea while 44% believe that ORS can aggravate diarrhea. Fifty nine point five percent (59.5%) of the mothers believe that ORS replaces fluid and electrolyte loss by diarrhea. Nearly half (44.1%) of study participants agreed that giving extra fluid during diarrheal episodes is necessary while a quarter (24.2%) of mothers disagreed with the statement. Since the data were fairly distributed as observed in a histogram mean was used in an assertion of the attitude of the respondents. In this study of the total respondents, more than half (55.5%) of the mothers had a favorable attitude while 44.5% had an unfavorable attitude.

### Factors associated with knowledge and attitude of mothers/caregivers towards management of diarrhea for under-five children

To identify factors associated with the knowledge and attitude of mothers on diarrheal management logistic regression was used with 95% CI and p-value < 0.05. All variables that have an association by binary logistic regression were entered into multiple-logistic regression and final independent predictors were identified. Age, relation to the child, marital status, and family size had no significant association with knowledge of mothers towards management of diarrhea by binary and multiple regression analysis. Educational status, occupation, monthly income, marital status, TV owners, and TV frequency had a significant association with knowledge of mothers/caregivers on diarrheal management at bivariate regression analysis. Multivariate analysis showed that only the educational status of mothers had a significant association with knowledge of mothers towards home-based management of diarrhea. Illiterate mothers were 88% times less likely to have good knowledge of diarrheal management as compared to college and above (AOR (95%CI) 0.12(0.027-0.554)) (Table 2). According to this study's educational status, the mother's relation to the child, monthly income, and age was found to have a significant association with the attitude of mothers toward the management of diarrhea at binary regression analysis. The multivariate analysis result showed relationship between the child and the monthly income of mothers had a significant association with the attitude of mothers towards home-based management of diarrhea. Participants who are mothers were 3 times more likely to have a favorable attitude towards the management of diarrhea as compared to those who are caregivers (AOR (95%CI) 3.085(1.071-8.890)). Mothers who earn < 2000 are 0.25 times less likely to have a favorable attitude towards the management of diarrhea as compared to those who earn > 4000 (AOR (95%CI)0.248(0.069-0.892)) (Table 3).

**Table 2:** factors associated with knowledge of mothers on home-based management of diarrhea among mothers/caregivers in HUSCH, Hawassa, Ethiopia, 2021

Variable	Knowledge		COR(95%CI)	AOR(95%CI)	P-value
	**Good**	**Poor**			
**Educational status Illiterate**	5(12.2%)	36(87.8%)	0.117(0.037-0.369)	0.123(0.027-0.554)	0.006**
**Read and write**	7(43.8%)	9(56.2%)	0.655(0.199-2.155)	1.225(0.274-5.484)	0.791
**Primary**	15(25.4%)	44(74.6%)	0.287(0.1189-0.697)	0.511(0.144-1.822)	0.301
**Secondary**	37(48.7%)	39(51.3%)	0.799(0.358-1.783)	1.165(0.391-3.471)	0.783
**College and above**	19(54.3%)	16(45.7%)	1	1	----
**Occupation Government employee**	12(57.1%)	9(42.9%)	0.2.246(0.798-6.315)	2.338(0.570-9.590)	0.238
**Self-employee**	25(43.1%)	33(56.9%)	1.276(0.591-2.755)	1.521(0.543-4.258)	0.425
**House wife**	27(27.8%)	70(72.2%)	0.650(0.316-1.336)	1.146(0.407-3.227)	0.796
**Merchant**	19(37.3%)	32(62.7%)	1	1	
**Monthly income <2000**	16(29.1%)	39(70.9%)	0.538(0.225-1.289)	1.271(0.380-4.247)	0.697
**2000-2999**	30(33.3%)	60(66.7%)	0.656(0.300-1.438)	0.784(0.264-2.335)	0.663
**3000-3999**	21(46.7%)	24(53.3%)	1.148(0.479-2.756))	1.716(0.599-4.914)	0.315
**>4000**	16(43.2%)	21(56.8%)	1	1	
**Marital status Married**	48(31.6%)	104(68.4%)	1.154(0.475-2.805)	0.970(0.356-2.639)	0.952
**Ummarried**	14(63.6%)	8(36.4%)	4.375(1.325-14.446)	3.597(0.874-14.808)	0.076

**Table 3:** factors associated with the attitude of mothers on home-based management of diarrhea among mothers/caregivers in HUSCH, Hawassa, Ethiopia, 2021

Variable	Attitude		COR(95%CI)	AOR(95%CI)	P-value
	Favorable	Unfavorable			
**Educational status**					
Illiterate	23(56.1%)	18(43.9%)	0.511(0.196-1.333)	1.566(0.319-7.683)	0.581
Read and write	7(43.8%)	9(56.2%)	0.311(0.091-1.065)	0.974(0.181-5.239)	0.975
Primary	23(39.0%)	36(61.0%)	0.256(0.104-0.629)	0.993(0.243-4.056)	0.992
Secondary	48(63.2%)	28(36.8%)	0.686(0.288-1.635)	2.402(0.650-8.873)	0.189
College and above	25(71.4%)	10(28.6%)	1		
**Relationship with child**					
Mother	101(58.0%)	73(42.0%)	3.360(1.325-8.518)	3.085(1.071-8.890)	0.037**
Grandmother	18(62.1%)	11(37.9%)	3.974(1.250-12.632)	2.284(0.547-9.539)	0.257
Caregiver	7(29.2%)	17(70.8%)	1		
**Monthly income**					
<2000	19(34.5%)	36(65.5%)	0.286(0.119-0.685)	0.248(0.069-0.892)	0.033**
2000-2999	49(54.4%)	41(45.6%)	0.647(0.293-1.430)	0.483(0.150-1.560)	0.224
3000-3999	34(75.6%)	11(24.4%)	1.674(0.642-4.364)	1.569(0.489-5.037)	0.449
>4000	24(64.9%)	13(35.1%)	1		
Age 15-24	17(29.8%)	40(70.2%)	0.597(0.203-1.755)	0.778(0.208-2.908)	0.709
25-34	49(38.3%)	79(61.7%)	0.375(0.138-1.020)	0.418(0.124-1.412)	0.160
35-44	7(35.0%)	13(65.0%)	0.458(0.127-1.660)	0.353(0.068-1.830)	0.215
45 and above	10(45.5%)	12(65.0%)	1		

## Discussion

Knowledge and attitude of mothers/caregivers are crucial for the management of diarrhea, especially for under-five children to prevent impact on their life. According to this study, 36.6% of mothers/caregivers had good knowledge on home-based management of diarrhea. This finding is lower than studies conducted in Nigeria 75.1% and Iran 65% [5,15]. Similarly in Ethiopian studies conducted in the Awi zone (56.2%) Finote Selam (63.6%) and Ginchi (63.8%) and Mareka (67%) revealed a higher level of knowledge than this study [14,16-18]. The possible justification for this discrepancy can be because the hospital where this study is conducted is a tertiary hospital participant involved in the study who came from far and rural pastoralist southern Ethiopia communities. This, in turn, can be related to the limited information available, and the difference in culture and lifestyle of northern and, central Ethiopia. Besides, the differences can be since this study has included not only mothers but also any caregiver spending a long time with a child, which is not commonly seen in other previously conducted studies. On the other hand, the level of knowledge found in this study is comparable with the 37.5% level of knowledge found in the study in the Asosa district. On the contrary, the finding in our study is higher than the study conducted in Iran with 28% levels of knowledge. [15,19,20]. These differences can be attributed to socio-demographic, cultural, and health-seeking behavior differences between the countries in general. Generally, knowledge of mothers/caregivers on home-based management of diarrhea is low and unacceptable. Knowledge is a baseline and a precursor for action. Since most of the management of a child with diarrhea is at the home level mothers who have a low level of knowledge about the management of diarrhea at the home level will have altered practice in a child's management of diarrhea. Which intern plays a negative role in the early and general prognosis of a child with diarrhea.

This study found that 55.5% of mothers/caregivers had a favorable attitude toward home-based management of diarrhea. This finding is higher than the findings of studies conducted in, Finote Selam, northwest Ethiopia, Dire Dawa, eastern Ethiopia, and Temeke Municipality, Tanzania 50.1%, 45.1%, and 19% respectively [8,16,20]. On the other hand, the finding is lower than the findings of studies conducted in Ginchi, Oromia, and Assosa western Ethiopia 62%, 62.9% respectively [17,19]. This discrepancy may be due to the sample size of the study. Attitude and perception of people are mainly influenced by culture, religion, and educational status of the community and individuals [21]. Since the above-mentioned studies are conducted in different areas throughout different communities with different cultures and educational statuses differences in the levels of attitude have been observed. Even though a better percentage of mothers have a favorable attitude towards home-based management of diarrhea compared to those who have a good knowledge it is still low. Most of the mothers/caregivers have a positive attitude toward the directions and management given by the health institutions. Increasing the mother's attitude will make the mother be curious about the management and follow the instructions given by the clinicians.

In this study relationship with the child had a significant association with the attitude of mothers towards home-based management of diarrhea. This might be because the mother will tend to give more attention and favor their children's home-based treatment in comparison to caregivers who are not mothers of the children. On the other hand, this study found that those mothers with low monthly income are less likely to have a favorable attitude compared to those mothers with the highest income. A similar study conducted in bench Maji zone, southern Ethiopia found out that the wealth index of the mothers is an independent predictor of mothers' perception of the diarrheal disease of children [22]. The relation of income with the mother's attitude goes hand in hand with educational status, this is because mainly in developing countries income increases with educational status [23]. Educational status, in turn, is related to the knowledge of the mother on home-based management of diarrhea. Besides the other justification can be, since those will low income are less likely to attend health institutions they might be unfamiliar with home-based management of diarrhea. The study used a crossectional study design, which doesn't show a causal relationship between the dependent variable and the predictors, which is a limitation. Besides the design study was purely qualitative, which limits exploration of the opportunities and obstacles in the study subject. On the other hand, the study used a pre-tested and validated tool which is a strength of the study.

## Conclusion

The knowledge and attitude of mothers/caregivers towards home-based management of their child´s diarrhea in this study is low. The educational status of mothers/caregivers is an independent predictor of knowledge of mothers/caregivers towards home-based management of diarrhea. Relationship with child and monthly income of mothers/caregivers had a significant association with attitudes of mothers/caregivers towards home-based management of diarrhea. The South nations nationalities peoples region and Sidama region health office shall work in Improving the educational status of mothers/caregivers and the federal as well regional financial sector shall emphasize raising the income of mothers which in turn plays a positive role in the improving knowledge and attitude of mothers towards home-based management of diarrhea.

### What is known about this topic

- *Mothers/caregivers play an important role in the home management of diarrhea;*
- *Mothers' level of literacy plays an important role in the home-based management of diarrhea*.

### What this study adds

- *This study has included both caregivers and mothers of children;*
- *That brings information about the differences in the knowledge and attitude towards home-based management of diarrhea between mothers and caregivers of children;*
- *This study shows that mothers have a 3fold more positive attitude towards home-based management of diarrhea compared to caregivers*.
